# Spike-Triggered Covariance Analysis Reveals Phenomenological Diversity of Contrast Adaptation in the Retina

**DOI:** 10.1371/journal.pcbi.1004425

**Published:** 2015-07-31

**Authors:** Jian K. Liu, Tim Gollisch

**Affiliations:** 1 Department of Ophthalmology, University Medical Center Göttingen, Göttingen, Germany; 2 Bernstein Center for Computational Neuroscience Göttingen, Göttingen, Germany; Université Paris Descartes, Centre National de la Recherche Scientifique, FRANCE

## Abstract

When visual contrast changes, retinal ganglion cells adapt by adjusting their sensitivity as well as their temporal filtering characteristics. The latter has classically been described by contrast-induced gain changes that depend on temporal frequency. Here, we explored a new perspective on contrast-induced changes in temporal filtering by using spike-triggered covariance analysis to extract multiple parallel temporal filters for individual ganglion cells. Based on multielectrode-array recordings from ganglion cells in the isolated salamander retina, we found that contrast adaptation of temporal filtering can largely be captured by contrast-invariant sets of filters with contrast-dependent weights. Moreover, differences among the ganglion cells in the filter sets and their contrast-dependent contributions allowed us to phenomenologically distinguish three types of filter changes. The first type is characterized by newly emerging features at higher contrast, which can be reproduced by computational models that contain response-triggered gain-control mechanisms. The second type follows from stronger adaptation in the Off pathway as compared to the On pathway in On-Off-type ganglion cells. Finally, we found that, in a subset of neurons, contrast-induced filter changes are governed by particularly strong spike-timing dynamics, in particular by pronounced stimulus-dependent latency shifts that can be observed in these cells. Together, our results show that the contrast dependence of temporal filtering in retinal ganglion cells has a multifaceted phenomenology and that a multi-filter analysis can provide a useful basis for capturing the underlying signal-processing dynamics.

## Author Summary

Our sensory systems have to process stimuli under a wide range of environmental conditions. To cope with this challenge, the involved neurons adapt by adjusting their signal processing to the recently encountered intensity range. In the visual system, one finds, for example, that higher visual contrast leads to changes in how visual signals are temporally filtered, making signal processing faster and more band-pass-like at higher contrast. By analyzing signals from neurons in the retina of salamanders, we here found that these adaptation effects can be described by a fixed set of filters, independent of contrast, whose relative contributions change with contrast. Also, we found that different phenomena contribute to this adaptation. In particular, some cells change their relative sensitivity to light increments and light decrements, whereas other cells are influenced by a strong contrast-dependence of the exact timing of their responses. Our results show that contrast adaptation in the retina is not an entirely homogeneous phenomenon, and that models with multiple filters can help in characterizing sensory adaptation.

## Introduction

Sensory systems have to encode stimuli over wide input ranges, and neurons therefore adapt their processing characteristics to the encountered stimulus statistics. In the vertebrate retina, ganglion cells adjust their sensitivity and temporal filtering characteristics when visual contrast changes [1–9]. While several studies have identified different mechanisms that contribute to sensitivity changes of ganglion cells [9–13], the origins of the contrast-dependent changes in temporal filtering are much less understood. Early studies typically investigated changes in temporal filtering in the frequency domain by observing how the encoding of sinusoidal signals at different frequencies is affected by contrast [7,8,14]. More recent work [1–3,6,9] has shifted the focus towards measuring the filter characteristics in the time domain by using white-noise stimuli and computing the filter as the spike-triggered average (STA). In agreement with the frequency-domain studies, the STA analyses have shown that higher contrast leads to faster kinetics and a shift from low-pass to band-pass filtering characteristics.

Here, we take a new perspective on the contrast dependence of temporal filtering in the retina by taking multiple parallel filters into account. As a direct extension of the STA, spike-triggered covariance (STC) analysis allows the extraction of a set of multiple relevant filters from experimental data [15–19]. Indeed, STC and related analyses of retinal ganglion cells have typically revealed several relevant filters, corresponding to different stimulus features that influence the cell’s spiking response [20–25].

We therefore here ask how the set of multiple stimulus features is affected when visual contrast changes. For example, one expectation may be that the entire set of features shifts so that it covers different regions of stimulus space for different contrast levels. Alternatively, a fixed set of features may suffice to capture the relevant stimulus space across contrast levels, but the relative importance of the individual features may be altered, which then results in the contrast dependence of temporal filtering as measured by the STA. Based on multielectrode-array recordings from isolated salamander retinas, we investigated these possibilities by applying spike-triggered analysis, both STA and STC, to assess the temporal filtering characteristics and the set of relevant features under high and low visual contrast.

## Results

### Spike-triggered analyses of contrast adaptation

**Fig 1:**
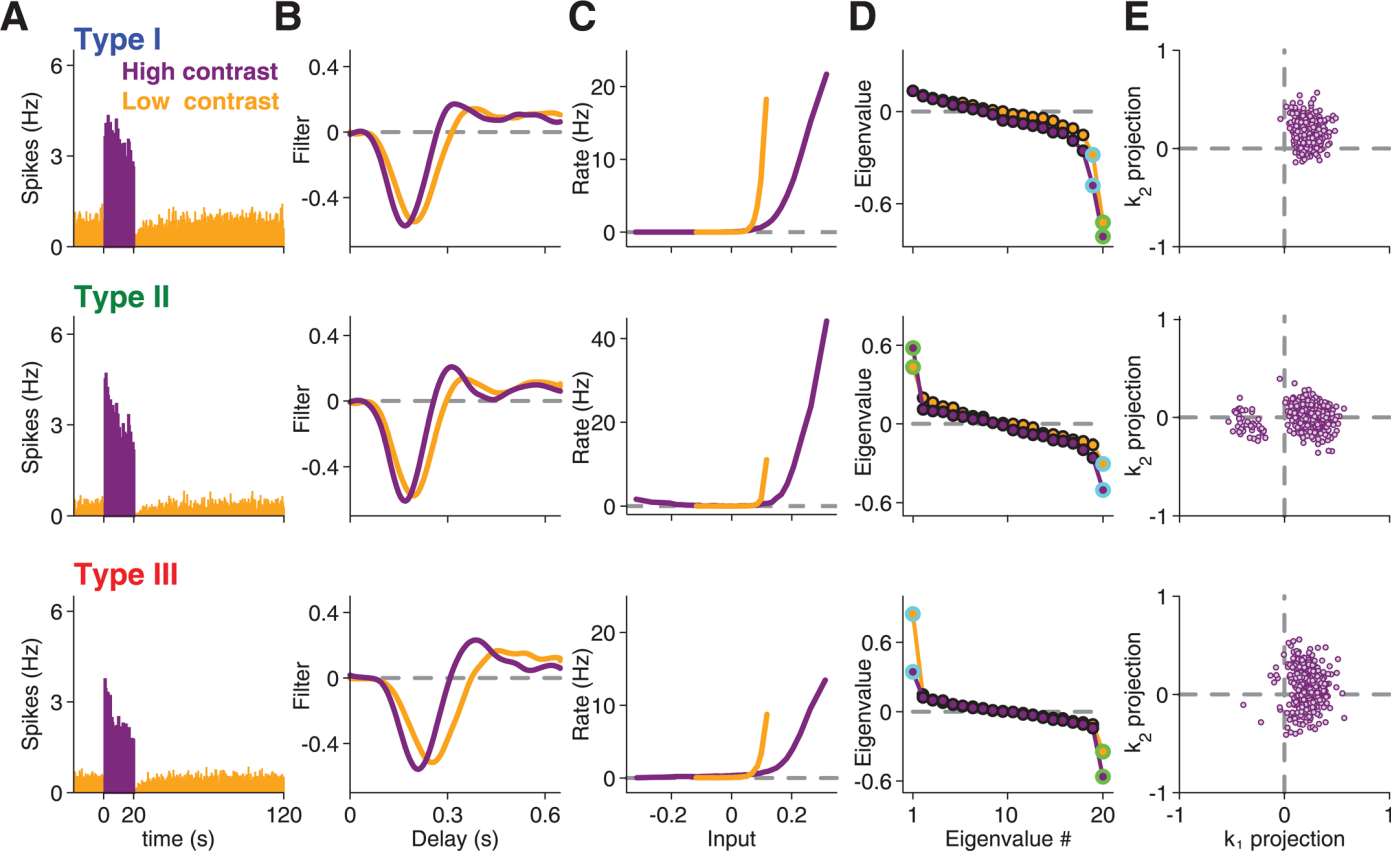
Spike-triggered analysis of contrast adaptation. Analyses for three sample cells, representative for Type I (top), Type II (middle), and Type III (bottom), under high contrast (purple) and low contrast (orange). (**A**) Firing rate histograms, averaged over all trials of high contrast (0–20 s) and low contrast (20–120 s). Note that the firing rates were obtained as trial averages with a different white-noise sequence in each trial. (**B**) Spike-triggered averages (STAs) for each contrast. (**C**) Corresponding nonlinearities for each contrast. Note that the slightly non-monotonic shape of the nonlinearity for the second sample cell reflects On-Off response characteristics of this cell [23]. (**D**) Eigenvalue spectrum obtained by STC analysis. The eigenvalues corresponding to the most and second-most informative features are marked by green and light blue circles, respectively. (**E**) Scatter plot of spike-triggered stimuli, projected onto the most informative stimulus feature *k*_1_ and the second-most informative feature *k*_2_. For clarity, only 10% of all analyzed spikes are shown in this plot.

In order to investigate how the visual features represented by a single ganglion cell are affected when contrast changes, we conducted multielectrode-array recordings from isolated salamander retinas. Visual stimuli consisted of a spatially uniform white-noise flicker of light intensity. The contrast level of this flicker stimulus was alternated between a low level (12%), lasting for 100 sec, and a high level (32%), lasting for 20 sec. The relatively longer presentation of low-contrast stimulation allowed us to collect comparable numbers of spikes under both conditions, despite the higher firing rates for high contrast (Fig 1A), thus providing the basis for reliable filter estimation at both high and low contrast. We restricted our analysis to cells with Off-type filter shapes, which represent the vast majority of ganglion cells in the salamander retina [26–28]. Note, though, that under full-field steps of light intensity, many of such Off-type ganglion cells in the salamander retina show On-Off response characteristics to various degrees [26,29,30].

We obtained temporal filters for each contrast level by computing the STAs (Fig 1B). The filters showed the typical changes known from other studies of contrast adaptation [1–3,6,9]; for higher contrast, the STA displayed a shorter time-to-peak and became more biphasic. Though we focus in this work on analyzing the filter changes, we also checked the contrast-induced sensitivity changes by computing the nonlinearities that describe the relation between the filter output and the resulting spike rate [31]. As expected, the nonlinearities were shifted to the right for higher contrast (Fig 1C), corresponding to reduced sensitivity.

In order to determine whether multiple stimulus features affected a ganglion cell’s response, we performed STC analysis for each contrast level. We collected spike-eliciting stimuli, defined as those stimulus segments that preceded a spike. We then computed the covariance matrix of these spike-eliciting stimuli and subtracted the prior stimulus covariance matrix. From this covariance matrix difference, relevant features are identified by an eigenvalue analysis [15–18,32]. In brief, an eigenvalue that is significantly different from zero means that, along a particular direction in stimulus space, stimulus segments that elicited spikes and those that did not are distributed differently. The corresponding stimulus direction, which can be identified by the corresponding eigenvector, therefore denotes a stimulus feature to which the cell is sensitive. As observed previously for salamander retinal ganglion cells [20], the obtained spectra of eigenvalues (Fig 1D) typically displayed two or more eigenvalues that deviated substantially from the baseline at zero, as also confirmed by statistical testing (see Methods). Indeed, under the high-contrast condition, none of the analyzed 345 cells revealed fewer than two significant eigenvalues, reconfirming that ganglion cell responses are affected by multiple visual features.

### Distinction of three response types based on STC analysis

**Fig 2:**
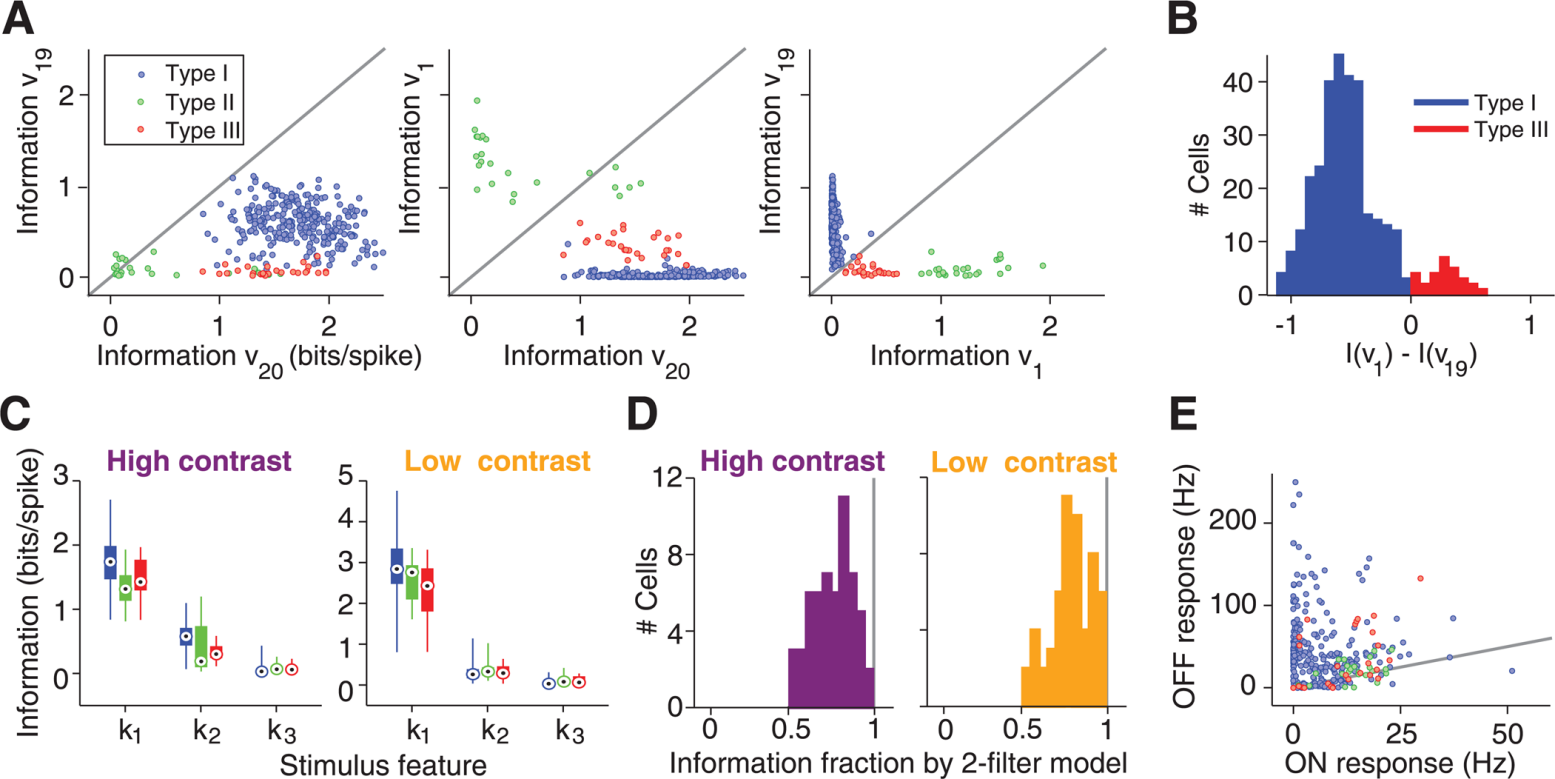
Information-theoretic analysis of relevant stimulus features. (**A**) Scatter plots of information rates carried by the features corresponding to the most positive (*v*_1_) and the two most negative (*v*_19_, *v*_20_) eigenvalues under high-contrast stimulation. (**B**) Histogram over all Type I and Type III cells of the difference in information rates for *v*_1_ and *v*_19_. (**C**) Distributions of information rates for the three most informative stimulus features, *k*_1_, *k*_2_, and *k*_3_, respectively, extracted from the STC analyses. The bar graphs denote the median by a circled black dot, the range between first and third quartile by a thick bar, and the entire range of information values by a thin line. (**D**) Histograms of the fraction of information captured jointly by *k*_1_ and *k*_2_, relative to the total information per spike. This analysis was performed only for a subset of cells where recordings with repeated white-noise sequences were available for measuring the total information. (**E**) Average firing rates for each cell to alternating bright (“ON response”) and dark (“OFF response”) illumination. The gray line depicts equality.

Similar to previous observations [20], we observed different types of eigenvalue spectra, in particular with respect to the occurrence of a significant positive eigenvalue (Fig 1D). To analyze the contrast dependence of the feature sets separately for different types of eigenvalue spectra, we sought a practically useful subdivision of our recorded cells. To do so, we obtained a measure of the importance of each identified feature by computing how much information it carried about the ganglion cell’s response (see Methods). We then compared the information values corresponding to the features from the high-contrast data with the most positive eigenvalue, *v*_1_, and with the two most negative of the 20 eigenvalues, *v*_19_ and *v*_20_ (Fig 2A).

The scatter of the information values supported the existence of different types of eigenvalue spectra, although the number of types and their exact boundaries are not entirely clear from this data. Yet, for the purpose of the subsequent analyses, we found the following classification to be useful. Type I cells were defined as those for which the two most informative features were *v*_19_ and *v*_20_ (see Fig 1, top row, for an example). These cells were by far the most frequently observed ones in our recordings, and they typically showed no substantial information in *v*_1_. By contrast, Type II cells were those for which *v*_1_ was particularly informative (Fig 1, center row), here defined as having an information rate of at least 0.8 bits/spike. Finally, Type III cells were defined as the cells in between, with a *v*_1_ feature that was less informative than those of Type II cells, but more informative than *v*_19_ (Fig 1, bottom row). Altogether, this procedure separated the analyzed cells into 293 cells of Type I, 24 cells of Type II, and 28 cells of Type III. Note that we do not claim that these types match actual ganglion cell types, and we cannot exclude that the data form a continuum. Type III cells, for example, border on the large group of Type I cells, yet an analysis of the differences in information values between *v*_1_ and *v*_19_ (Fig 2B) speaks against a single broad distribution and justifies treating Type III cells as a separate group.

The analysis of the information rates also showed that information deteriorated quickly after the first two most informative features (Fig 2C) and that the combination of the two most informative filters captured around 80% of the total information contained in the occurrence of individual spikes (Fig 2D), similar to previous findings [20]. Given the good performance of the two-feature model and the small information values of further features, we focused our subsequent analysis for each cell on the two most informative visual features. This choice allowed us to analyze all cells across both contrast levels in a unified fashion, despite the fact that the actual number of significant features depended sensitively on the number of analyzed spikes and on the cutoff used to determine significance and varied between cells and contrast levels. In the following, we denote the two most informative features by *k*_1_ and *k*_2_, respectively. For cells of Type I, *k*_1_ and *k*_2_ corresponded to *v*_20_ and *v*_19_, respectively, whereas *k*_1_ and *k*_2_ were defined as *v*_1_ and *v*_20_ for Type II cells and as *v*_20_ and *v*_1_ for Type III cells.

How the identified features relate to a cell’s spiking activity can be illustrated by projecting all spike-eliciting stimuli onto the two identified features *k*_1_ and *k*_2_ (Fig 1E). This shows that the positive eigenvalue for Type II cells corresponds to the occurrence of distinct clusters of spike-eliciting stimuli (Fig 1E, center row), whereas Type III cells display a continuously elongated region of spike-eliciting stimuli along the dimension of the positive eigenvalue (Fig 1E, bottom row). The distinct clusters of spike-eliciting stimuli, as found here for Type II cells, had previously been shown to correspond to spikes elicited by the On and the Off pathway, respectively [20,21,23,24,33]. Note that responses to full-field steps of light intensity did not distinguish these cells from the other two types because many cells in our recordings from all three types had some level of On-Off response characteristics under light-intensity steps (Fig 2E). Yet, the appearance of two clusters in the spike-eliciting stimuli suggested that cells of Type II have particularly strong and distinct On responses even under the applied flicker stimuli. This occurrence of two clusters of spike-eliciting stimuli was a defining characteristic of Type II cells; in fact, our criterion for separating between Type II and Type III cells based on the information of *v*_1_ assigned all cells with such clusters to Type II.

For each cell, the basic structure of the eigenvalue spectrum was generally similar for the high-contrast and low-contrast data (Fig 1D). In particular, the occurrence of a significant positive eigenvalue did not depend on the contrast level. Yet, closer inspection revealed subtle differences; in general, slightly more eigenvalues deviated significantly from the baseline at zero when contrast was higher, in particular for cells of Type I and II. These differences did not result from different spike numbers under the two contrast conditions, as they persisted when spike numbers were matched by discarding surplus spikes for either of the two contrast conditions. This yielded an average of 4.5±0.1 significant eigenvalues (mean±SEM) at high contrast and 4.1±0.1 at low contrast. To evaluate this further, we calculated for each cell the difference in the number of significant eigenvalues, ∆*EV*, between high and low contrast, taking into account equal numbers of spikes for both conditions. We found that there were more significant eigenvalues at high contrast for Type I cells (∆*EV* = 0.4±0.1, mean±SEM, p<10^−3^, Wilcoxon signed-rank test) as well as for Type II cells (∆*EV* = 0.7±0.2, p<10^−2^), but not for Type III cells (∆*EV* = 0.0±0.2, p>0.5).

### Reconstruction of STA with STC-derived features

Based on the STC analysis of the three distinguished cell types, we then asked whether the identified stimulus features provide a useful basis for explaining the contrast-induced changes in the STA. To approach this question, we explored whether the STA could be described as a linear combination of the features *k*_1_ and *k*_2_. In particular, we asked whether a single such two-feature basis is sufficient to describe the STAs from both contrast conditions. This would mean that for both contrast conditions the cell remained sensitive to the same stimulus subspace, spanned by the STC-derived features, and that only the relative contributions of the features changed under contrast adaptation. To avoid using the same spikes for computing the STAs as well as the features, we split the data for each contrast level into a training set, from which we obtained *k*_1_ and *k*_2_, and a test set, from which we computed the STA (see Methods).

**Fig 3:**
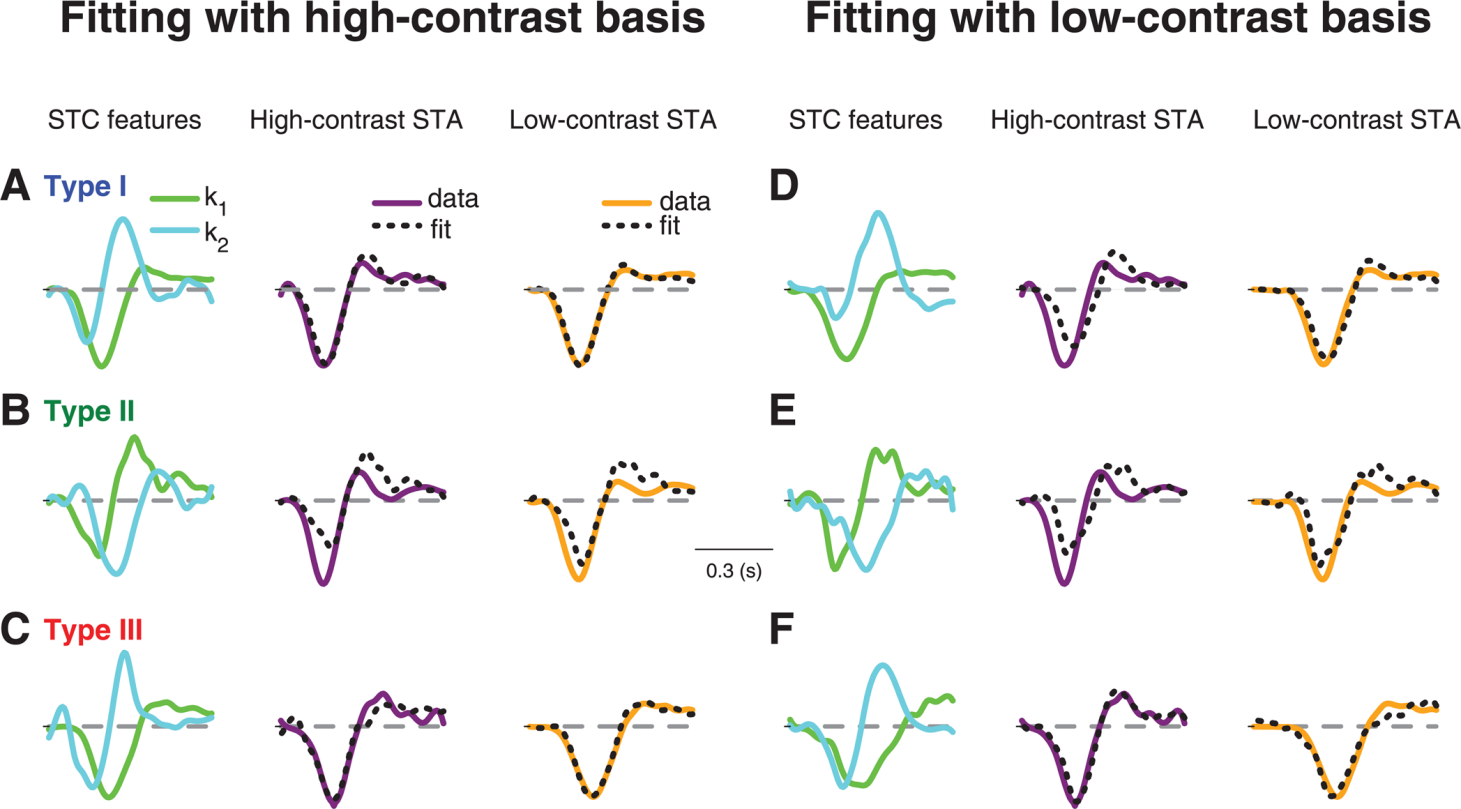
STA fits with STC-derived features for three sample cells. (**A-C**) Fits of high-contrast and low-contrast STAs with features derived from the high-contrast STC analysis. The three sample cells shown in (A), (B), and (C) are the same as in Fig 1. The STC-derived features *k*_1_ and *k*_2_ (left column) are used as a basis for fitting the high-contrast STA (middle column) and the low-contrast STA (right column). (**D-F**) Same as (A-C), but using *k*_1_ and *k*_2_ as derived from the low-contrast STC analysis.

Fig 3 shows how the features *k*_1_ and *k*_2_, obtained either under high contrast (Fig 3A, 3B and 3C) or under low contrast (Fig 3D, 3E and 3F), fitted the STAs from both contrast conditions for the three sample cells of Fig 1. Note that the STA fits with STC features derived from the same contrast level are expected to be good, as long as the relevant feature space is well described by just *k*_1_ and *k*_2_, because the STA is generally a linear combination of all relevant features [15,34]. The actual analysis of interest, on the other hand, is to fit the STAs with STC features derived from a different contrast level. If, for example, contrast adaptation alters the relevant feature space itself, these fits across contrast conditions should fail.

**Fig 4:**
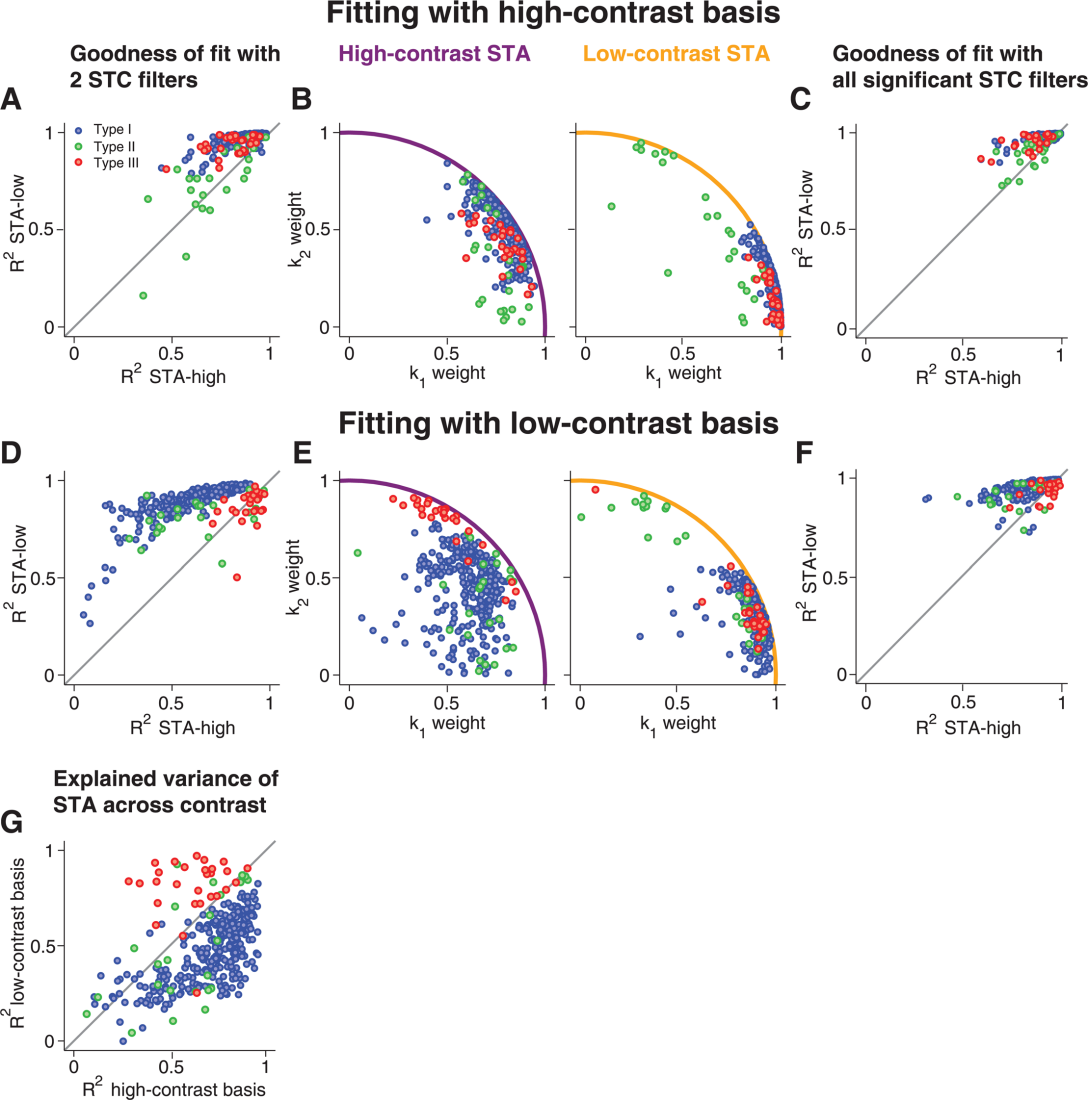
Population analysis of STA fits with STC-derived features. (**A**) Assessment of fit quality by the coefficient of determination *R* ^2^ for fitting high-contrast and low-contrast STAs with the high-contrast-derived features. The gray line marks identity. (**B**) Weights of *k*_1_ and *k*_2_ obtained from fitting the high-contrast STAs (left) and low-contrast STAs (right) by high-contrast-derived features. The colored lines mark the unit circle, which is a bound for the weights. (**C**) Same as (A), but using for each cell all features obtained as significant from the high-contrast STC analysis. (**D-F**) Same as (A-C), but based on the low-contrast-derived features for fitting the STAs. (**G**) Comparison of how much of the contrast-induced variation of the STAs were captured by the two-feature basis obtained from either high contrast or low contrast. This was quantified by the coefficient of determination, *R* ^2^, for the difference of the fitted STAs.

To evaluate the goodness of fit for the STAs of all recorded cells, we computed the coefficient of determination (*R* ^2^) for each fit (Fig 4A and 4D). This coefficient measures the fraction of variance in the STA that is captured by the fit, so that values near unity correspond to a perfect fit. We also inspected the distribution of the weights for *k*_1_ and *k*_2_ obtained from the fits (Fig 4B and 4E). Note that the weights implicitly also denote the fit quality: because the features and the STA are normalized and because the weights correspond to the projections of the STA onto the features, the radius of the data points in these plots is bounded by the unit circle, which is reached only if the STA is a linear combination of *k*_1_ and *k*_2_, i.e., if the fit is perfect.

For Type I cells, we found–as expected–that the high-contrast basis provided a good fit for the high-contrast STA, as shown by the sample cell (Fig 3A) as well as by the coefficients of determination (Fig 4A). Yet, the same basis, obtained from the high-contrast STC analysis, also provided an excellent fit for the low-contrast STA, showing that a single basis served to capture temporal filtering at both contrast levels. The good fits for both STAs were achieved with different contributions from the two features; the *k*_2_ feature became relatively more important for the high-contrast STA, as reflected by the change in weights for *k*_1_ and *k*_2_ (Fig 4B).

Surprisingly, the fit of the low-contrast STA with the high-contrast basis was even systematically better than the fit of the high-contrast STA with the same basis (Fig 4A). This implies that, while the high-contrast STC features provide a useful basis for either contrast level, additional features must become more relevant at high contrast. The low-contrast basis, on the other hand, provided a good fit only for the low-contrast STA, but not for the high-contrast STA (Figs 3D and 4D), indicating that the low-contrast basis lacks some of the structure that becomes relevant at high contrast.

For Type II cells, in contrast to Type I cells, the fits of the STAs were altogether poor; neither the high-contrast (Figs 3B and 4A) nor the low-contrast basis (Figs 3E and 4D) provided a good fit to either of the two STAs. This indicates that additional dynamics beyond those captured by the feature basis *k*_1_ and *k*_2_ are required to describe temporal filtering in these cells.

For Type III cells, the high-contrast basis generally provided good fits, similar to the case of Type I cells (Figs 3C and 4A). Unlike for Type I cells, however, the low-contrast basis for Type III cells also yielded good fits for both STAs (Figs 3F and 4D). This indicates that, for Type III cells, the relevant dynamics that mediate contrast adaptation are already present under low-contrast stimulation, in line with our earlier observation that the number of significant features did not increase with contrast for Type III cells, in contrast to the other two types.

To investigate the effect of using only two features in these fits, we also fitted the STAs with all features detected as significant in the STC analysis (Fig 4C and 4F). Naturally, fits improved as compared to using the two-feature basis. The improvement was particularly pronounced for Type II cells, confirming that additional dynamics beyond the first two features are important for capturing temporal filtering in these cells. Otherwise, the findings remained similar to the two-feature fits. In particular, for Type I cells, low-contrast STAs were still generally better fitted than high-contrast STAs, confirming that temporal filtering at high contrast is affected by a larger number of emerging filters, not all of which reach significance in the STC analysis.

The analyses above show that, for most cells, a single basis can fit the STAs from both contrast levels. In principle, this could result from STAs that just do not vary much with contrast so that a good fit at one contrast immediately implies a good fit at the other contrast. Thus, to check whether the features actually capture the variations of the STAs with contrast, we computed how much of the contrast-induced STA variance was explained by the fits with *k*_1_ and *k*_2_ (Fig 4G) by measuring the coefficient of determination for the difference of the STAs and the difference of their fits (see Methods). This analysis corroborated the distinction between Type I and Type III cells; for the former, the high-contrast basis captured the contrast-induced STA changes much more accurately, whereas the low-contrast basis was clearly superior for Type III cells.

### Contrast adaptation models show consistency with Type I, but not Type II or III cells

**Fig 5:**
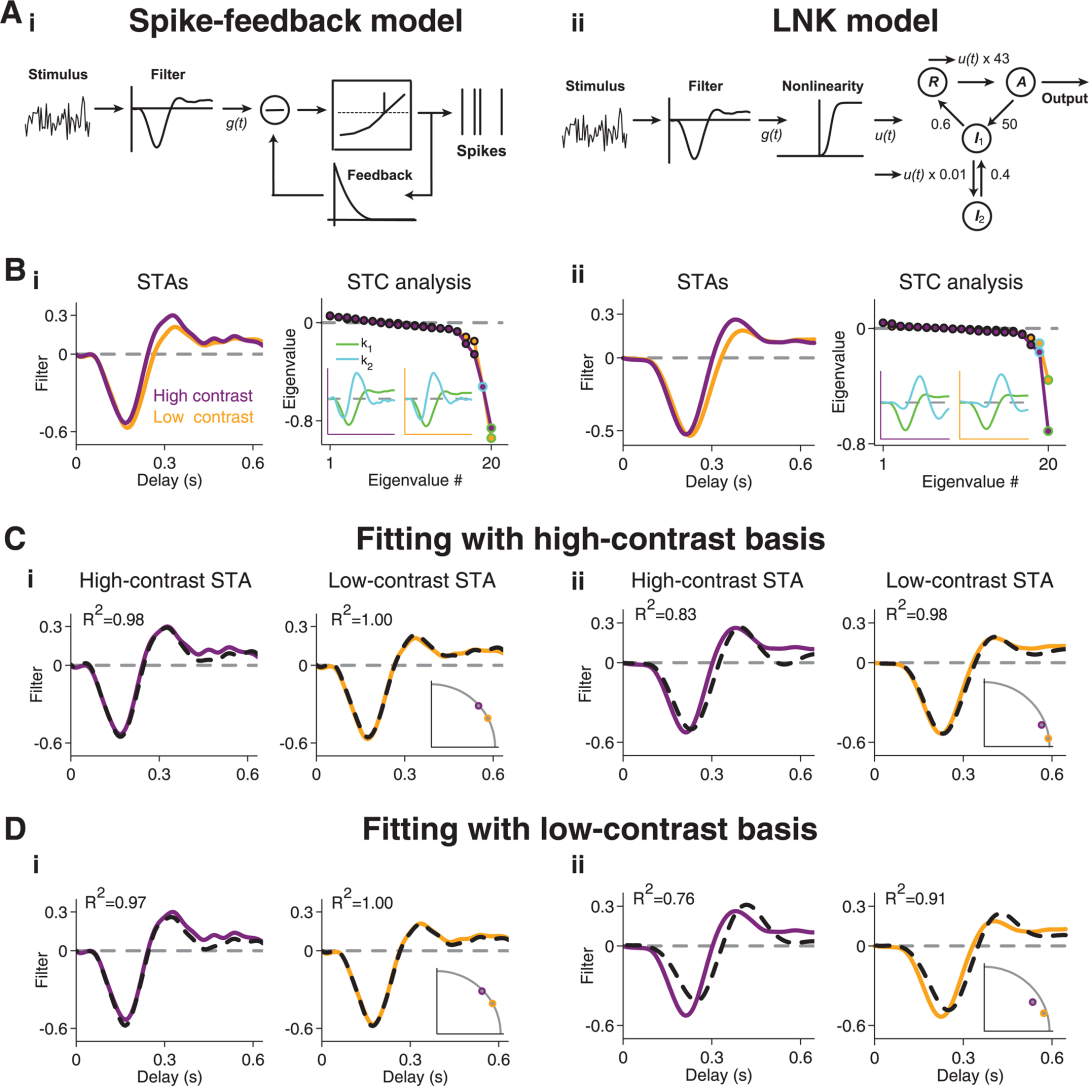
STC analysis of contrast adaptation models. (**A**) Layout of the models. (**i**) Spike-feedback model [35,36]. The model consists of a linear filter, a threshold operation to determine spikes, and an exponentially decaying feedback signal that is subtracted from the feedforward filter output after a spike has occurred. (**ii**) LNK model [37]. Adaptation is modeled by a kinetic model with a resting state R, an active state A, and two inactive states I_1_ and I_2_. Possible transitions between the states are indicated by arrows, together with the applied transition rates. The input to the kinetic model, u(t), is derived from a linear-nonlinear cascade and modulates the transition from the resting to the active state as well as from the first to the second inactive state. (**B**) STAs obtained from the two models under high- and low-contrast stimulation as well as corresponding STC spectra and features *k*_1_ and *k*_2_. (**C**) STA fits with features obtained from high-contrast STC analysis. The corresponding *R* ^2^ values are indicated in the plots. The inset shows the weights for *k*_1_ and *k*_2_ obtained from the fits, with the gray line indicating the unit circle and data points near the circle corresponding to good fits. (**D**) Same as (C), but for STA fits with features obtained from the low-contrast STC analysis.

In order to explore the origins of the observed differences between cell types further, we compared our results to computational models of contrast adaptation. Mechanistically, the primary source for contrast adaptation is thought to be activity-dependent gain control in the form of synaptic depression at bipolar cell terminals [9,12,13] and adaptation in the spike generation mechanism of the ganglion cell [10,11]. We therefore performed the spike-triggered analyses on two established models that contain such activity-dependent gain control in order to check which of the three distinguished cell types are consistent with these models or require other dynamics. Both models have previously been shown to accurately capture many phenomena of contrast adaptation, in particular with respect to the observed changes in temporal filtering. The first model [35], termed here spike-feedback model, is based on a linear temporal filter, followed by a threshold evaluation to obtain spikes, together with negative feedback that is subtracted from the filtered signal after each spike (Fig 5Ai). The model can be used to predict individual spikes of retinal ganglion cells [35] and has been shown to capture the phenomenology of contrast adaptation surprisingly well [36]. The second model [37], termed LNK model, is based on a linear-nonlinear (LN) cascade, composed of a linear temporal filter and a nonlinear transformation, which is followed by a first-order kinetic process (Fig 5Aii, Methods). The kinetic process matches well the dynamics of synaptic depression at bipolar cell terminals, and the model has been shown to provide an accurate account of contrast adaptation at the level of the ganglion cell membrane potential [37].

When stimulated with high- and low-contrast white noise, both models showed changes in temporal filtering (Fig 5B) similar to experimental observation; STAs became narrower and more biphasic at high contrast. In both cases, STC analysis revealed significant negative eigenvalues, but no significant positive eigenvalue, reminiscent of Type I cells. We then analyzed how the two STAs could be fitted by the STC-derived features. For the spike-feedback model, differences between the STAs and STC-derived features were small, and good fits were obtained in all cases. For the LNK model, the STA fits showed similar behavior as observed for Type I cells. Only the high-contrast basis provided good fits for both STAs (Fig 5Cii and 5Dii), with a better fit for the low-contrast STA. Like for Type I cells, the latter follows from the growing importance of more than just the first two features at higher contrast (Fig 5Bii).

Thus, these gain-control models are consistent with our findings for Type I cells, but not for the other two types. In particular, the models did not produce a positive eigenvalue, even when we varied model parameters over considerable ranges. This appears to be a generic feature, for which the following reasoning provides some intuition: The core element of the models is a single temporal filter followed by a threshold or a fairly steep nonlinearity, necessary to account for the experimentally observed response sparseness. Thus, only sufficiently strong activation of the filter triggers spikes, leading to a reduced variance of the spike-triggered ensemble along this stimulus dimension, corresponding to a negative eigenvalue. The negative-feedback dynamics in the models provide suppression of spikes, leading to reduced variance in the spike-triggered ensemble and thus further negative eigenvalues for the corresponding suppressive filters [15,38,39]. Therefore, we will in the following consider other dynamics for capturing the contrast dependence of temporal filtering in Type II and Type III cells.

### STC analysis for Type II cells with separation into On- and Off-pathway signals

**Fig 6:**
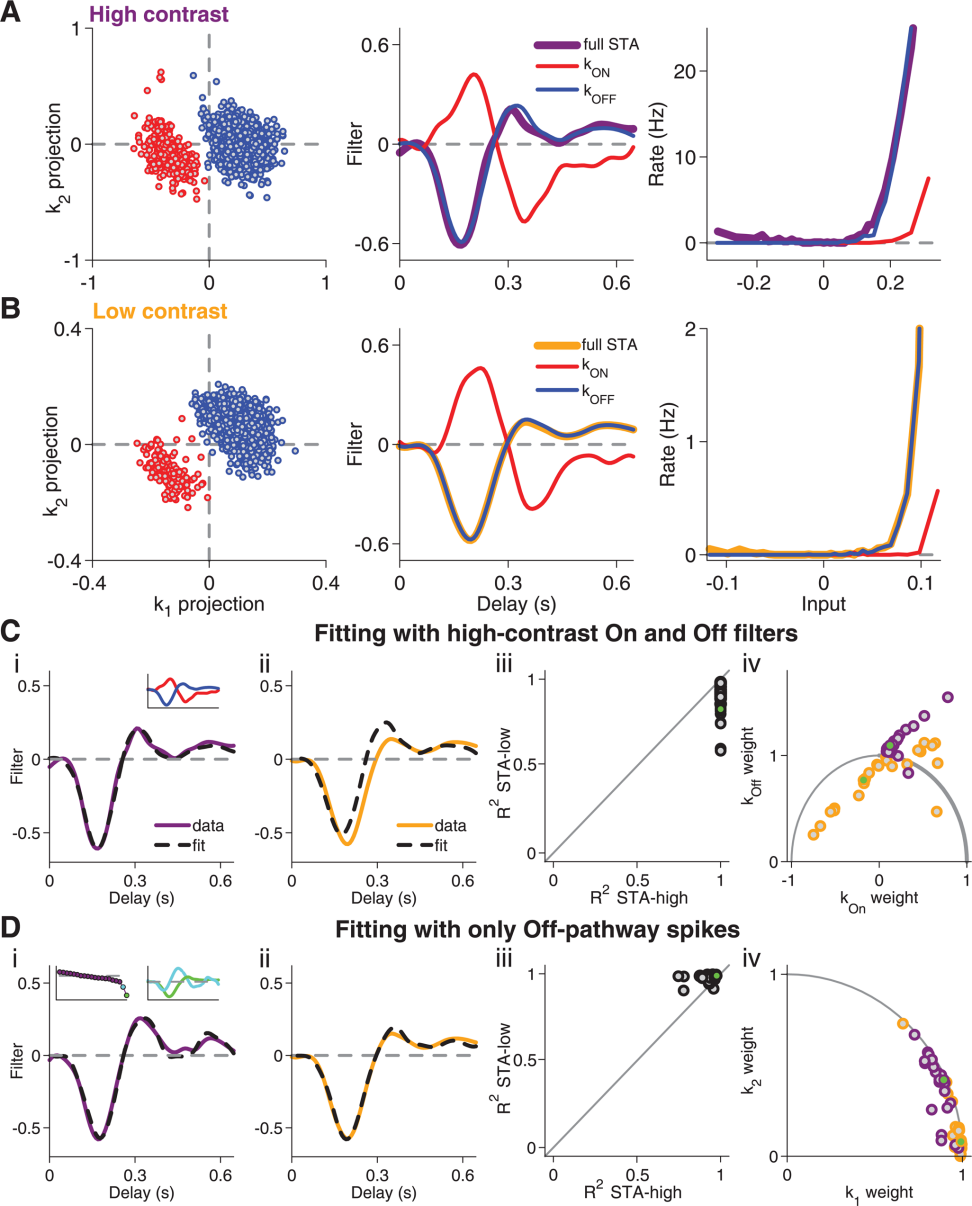
Analysis of contrast-dependent filter changes in Type II cells after separation of On and Off pathways. (**A**) Separation of spike-triggered stimuli into On and Off pathway shown for a sample cell of Type II. Left: Scatter plot of spike-triggered stimuli for high-contrast stimulation, projected onto *k*_1_ and *k*_2_. Red and blue data points mark the clusters corresponding to the On and Off pathways, respectively. Center: Filters *k*_*ON*_ and *k*_*OFF*_, obtained by computing the STAs for the two clusters separately, as well as the full STA, obtained from all spikes. Right: Nonlinearities obtained from the separated On and Off clusters (red and blue, respectively) as well as for the entire set of spikes (thick line). (**B**) Same as (A), but for low-contrast stimulation. (**C**) STA fits with the *k*_*ON*_ and *k*_*OFF*_ features, obtained under high contrast. Fit of high-contrast STA (**i**) and low-contrast STA (**ii**) for the sample cell as well as coefficients of determination (**iii**) and corresponding weights obtained for the two features (**iv**) for all recorded cells of Type II. Note that, in contrast to *k*_1_ and *k*_2_, *k*_*ON*_ and *k*_*OFF*_ are generally not orthogonal to each other. Thus, the sum of squared weights obtained from the fit is not bounded by unity. The inset in (i) shows *k*_*ON*_ and *k*_*OFF*_. The green data points in (iii) and (iv) mark the sample cell. (**D**) Same as (C), but using only spikes from the Off-pathway cluster and their features *k*_1_ and *k*_2_, derived from the high-contrast STC analysis. The insets in (i) show the corresponding eigenvalue spectrum as well as *k*_1_ and *k*_2_.

STAs of Type II cells were generally poorly fitted when using the two features derived from the STC analysis. To investigate this cell type further, we come back to the finding that Type II cells exhibited two clusters of spike-eliciting stimuli (Fig 1E), which had previously been attributed to represent inputs from both On and Off pathways [20,21,23,24,33]. We therefore separated the two clusters (see Methods) and analyzed their spikes separately to compute STAs and corresponding nonlinearities for each cluster at both high contrast (Fig 6A) and low contrast (Fig 6B). For each contrast, the two STAs yielded filter shapes that are characteristic for the On and Off pathway, respectively, including the known relative delay of the On-type filter [24]. Furthermore, while the original nonlinearity showed a slightly non-monotonic shape, indicative of two contributing pathways [23], the separate analysis of the two clusters yielded monotonically increasing nonlinearities. These findings supported the notion that the filters of the two clusters represent inputs through the On and Off pathway, and we therefore termed the filters *k*_*ON*_ and *k*_*OFF*_.

The cells were dominated by the Off pathway; the corresponding cluster always contained many more spikes than that of the On pathway. The relative contribution of the On pathway, however, depended on the contrast level; at high contrast, a larger percentage of spikes was part of the On cluster (20±3% at high contrast, 8±2% at low contrast, p<10^−3^, Wilcoxon signed-rank test). Thus, contrast controls the relative effectiveness of the On and Off pathway in these cells, thereby influencing temporal filtering and how the filtered signals relate to the cell’s activity.

To check whether these changes in contributions from the two pathways are sufficient to explain the contrast dependence of temporal filtering in these cells, we asked whether a single set of filters *k*_*ON*_ and *k*_*OFF*_ can fit the STAs from both contrast conditions. Not surprisingly, the On and Off filters from the high-contrast data provided excellent fits of the STAs from the same contrast level (Fig 6C); by construction, the STA is a weighted average of the STAs from the two clusters. However, the fits of the low-contrast STAs with the high-contrast On and Off filters were typically poor and led to negative weights for the On filter (Fig 6C), inconsistent with the supposed representation of the physiological On pathway. Analogously, the low-contrast On and Off filters provided good fits only for the low-contrast STAs.

This analysis indicated that contrast-dependent changes in stimulus filtering in these cells does not only follow from modified relative contributions of the On and Off pathways. We therefore hypothesized that, in addition, the two pathways adapt individually. To test this hypothesis, we performed spike-triggered analysis only for those spikes of Type II cells that were part of the Off-pathway clusters. The STC analysis of these spikes showed that the Off pathway of Type II cells resembled a Type I cell, with all significant eigenvalues being negative (inset of Fig 6Di). Furthermore, the Off-pathway STAs from both contrast conditions were now well fitted by the two most relevant features obtained from the high-contrast STC analysis (Fig 6D). Also, the high-contrast STC features provided a better fit of the low-contrast than of the high-contrast STA, as had been the case for Type I cells.

We also attempted to perform a similar analysis with the spikes from the On-pathway cluster, but the much lower number of spikes contributed by this pathway rendered the STC analysis too noisy for a reasonable analysis of STA fits. Nonetheless, our findings suggest that the contrast-dependent changes in temporal filtering of Type II cells follow from independently adapting On and Off pathways, with the alterations in their relative contributions resulting from stronger adaptation in the Off pathway. This finding is in line with the previously reported stronger contrast adaptation in Off-type as compared to On-type ganglion cells in the salamander retina [3], suggesting that in general the Off pathway adapts more strongly in this system.

### Spike timing in Type III cells is affected by activation-dependent latency shifts

For Type III cells, the positive eigenvalue was not associated with the occurrence of distinct clusters of spike-triggered stimuli. Furthermore, Type III cells differed from the other two cell types in that they allowed particularly good fits of the high-contrast STA by the low-contrast STC features and did not increase the number of relevant features at higher contrast. This suggests that contrast adaption of temporal filtering is less associated with additional emerging features. We therefore looked for other dynamics that might be of particular importance for these cells.

Theoretical studies had shown that the occurrence of positive eigenvalues in the STC analysis together with an elongated distribution of spike-eliciting stimuli, as we had found for Type III cells, occur for the Hodgkin-Huxley model and are associated with effects on the precise timing of spikes [40]. A previous study [20] had hypothesized that positive STC eigenvalues observed in some retinal ganglion cells may similarly arise through such spike-timing dynamics. Furthermore, spike-time jitter and spike-latency shifts are known to affect temporal filters and eigenvalue spectra of spike-triggered analyses [41–43]. For studying contrast adaptation, such spike-timing dynamics may be particularly relevant, as contrast has a strong effect on the amount of jitter [44,45] as well as on spike latency [46–49].

**Fig 7:**
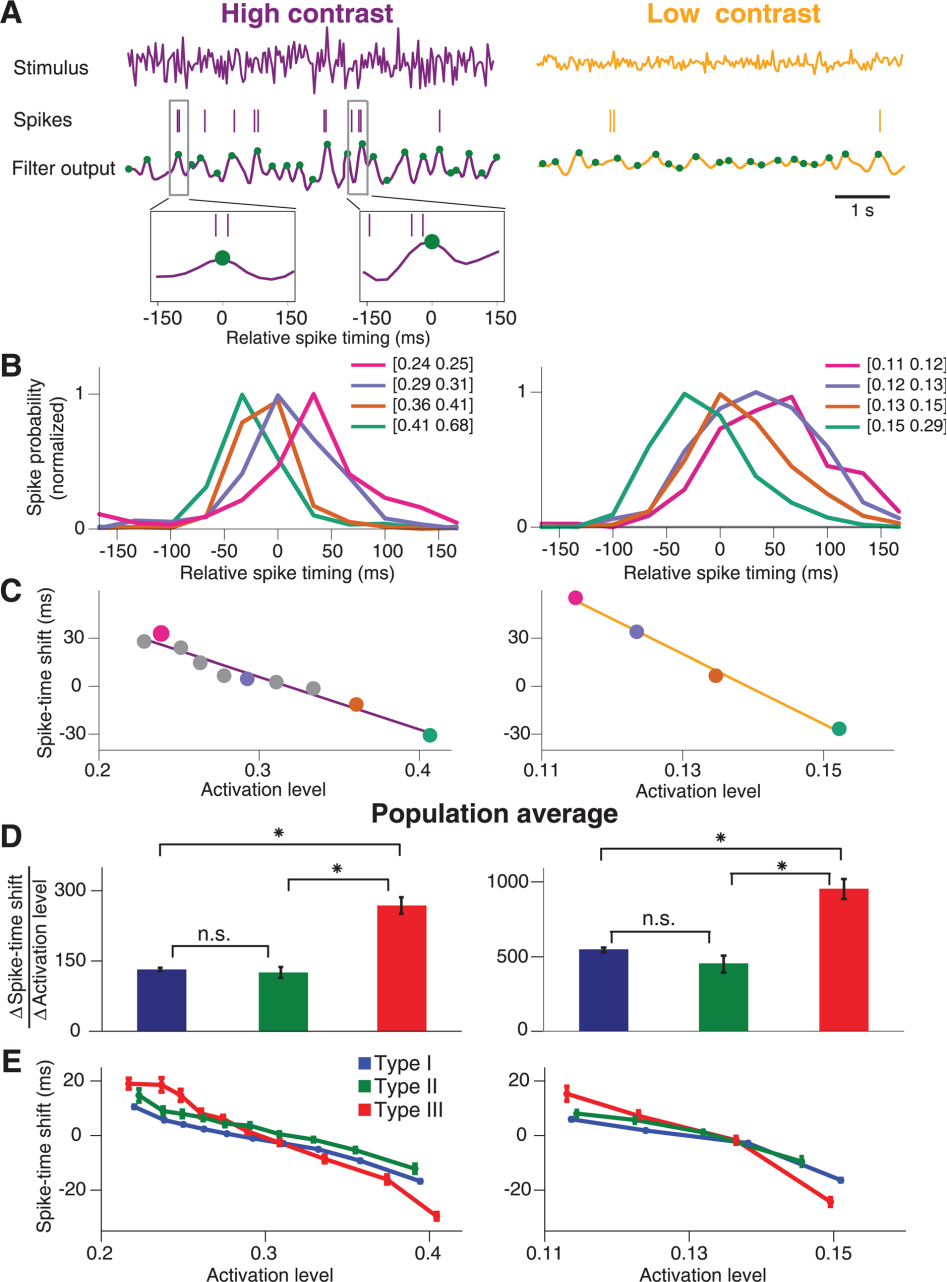
Estimation of spike-timing dynamics. (**A**) Stimulus segments of high contrast (left) and low contrast (right) and corresponding spike trains of a sample ganglion cell (here Type III). The cell’s filter output, obtained by convolving the stimuli with the cell’s corresponding STAs, is shown below. Local maxima are marked by green dots. Two segments of the filter output trace and corresponding spikes are shown enlarged to illustrate the collection of relative spike times in a window around local maxima. (**B**) Histograms of relative spike timing for peak values of the filter output in four different ranges. The boundaries of these ranges are specified in the legend. Histograms are normalized to their peak values. Note that the negative values of relative spike timing do not imply acausal effects. Rather, the computation of the activation level by filtering the stimulus with the STA includes an average delay between the relevant stimulus and the elicited spikes, in the range of around 100 ms (see, for example, the STA peak times in Fig 1B). The relative spike timing observed here can thus be viewed as a correction to this average delay. (**C**) The characteristic spike-time shift, computed as the location of the maximum of the relative spike-timing histogram, plotted versus the mean filter output peak size in each applied filter output range. The characteristic spike-time shifts become more negative with increasing filter output peak size, showing that stronger activation leads to relatively earlier spikes. The data points corresponding to the histograms depicted in (B) are marked by dots in the respective colors. Solid lines display linear fits to the data. (**D**) Population averages of the absolute values of the slopes (∆Spike-time shift/∆Activation level) obtained from the linear fits as illustrated in (C) for Type I (blue), Type II (green), and Type III (red) cells. Error bars denote standard errors. Differences between the populations are marked as significant (t-test at 5% significance level, Bonferroni corrected) by asterisks and otherwise by “n.s.” (**E**) Population average of the curves shown in (C) for Type I (blue), Type II (green), and Type III (red) cells. Error bars denote standard errors. The plot shows that Type III cells span a much larger range of delays than the other two types.

We therefore hypothesized that Type III cells are cells with particularly strong spike-timing dynamics. In particular, activity-dependent shifts in spike latency might play an important role for the contrast dependence of temporal filtering because such shifts can lead to delays in the filters, consistent with our experimental observations (Fig 1B), whereas jitter primarily broadens the filters. We therefore analyzed whether there exists a systematic effect of the stimulus on spike latency. For our white-noise stimuli, even at fixed contrast, the activation level of a ganglion cell fluctuates over time, and some spikes are elicited when the activation of the cell is strong while others result from episodes of smaller activation level. Thus, we aimed at assessing the relationship between activation level and timing of associated spikes for each ganglion cell. To obtain an estimate of the activation level, we used the STA of the cell to filter the stimulus sequence and then detected the peaks in this filter output (Fig 7A), as these peaks likely play a major role in generating spikes. We then gathered all spikes in the vicinity of each peak and constructed histograms of relative spike timing for different ranges of the activation level (Fig 7B).

This analysis indeed revealed a strong effect of activation level on spike timing. First, spike time histograms were broader at low contrast than at high contrast, indicating that spike time jitter was larger at low contrast. Second, for both high and low contrast, stronger activation levels led to relatively earlier spikes, indicating that spike latency directly depended on the activation level. These effects of activation level on spike jitter and on spike latency were observed for cells of all three types. We then tested whether these spike timing dynamics quantitatively differed between the cell types.

To do so, we quantified the effect of the activation level on the spike latency by extracting the relative spike timing for which each of the histograms reached its maximum. We then plotted this spike-time shift against the mean activation level for the corresponding histogram (Fig 7C). These plots generally revealed a continuous, approximately linear decrease of the spike-time shift with increasing activation level. For each cell, we then computed the slope of the spike-time shift versus the activation level and compared the slope values between cell types (Fig 7D). For both high and low contrast, this analysis showed that Type III cells indeed displayed a much stronger dependence of spike timing on activation level than either Type I cells or Type II cells (p<10^−6^ in all cases, t-test). In fact, the stimulus-induced shifts in spike timing were about twice as big for Type III cells as compared to the other two types. The stronger dependence of spike timing on activation level in Type III cells can also be illustrated by averaging the dependence of spike-time shifts on activation levels over all cells of a given type (Fig 7E), showing that Type III cells span a bigger range of spike-time shifts than the other two types.

### Latency-shift model explains contrast dependence of temporal filtering in Type III cells

**Fig 8:**
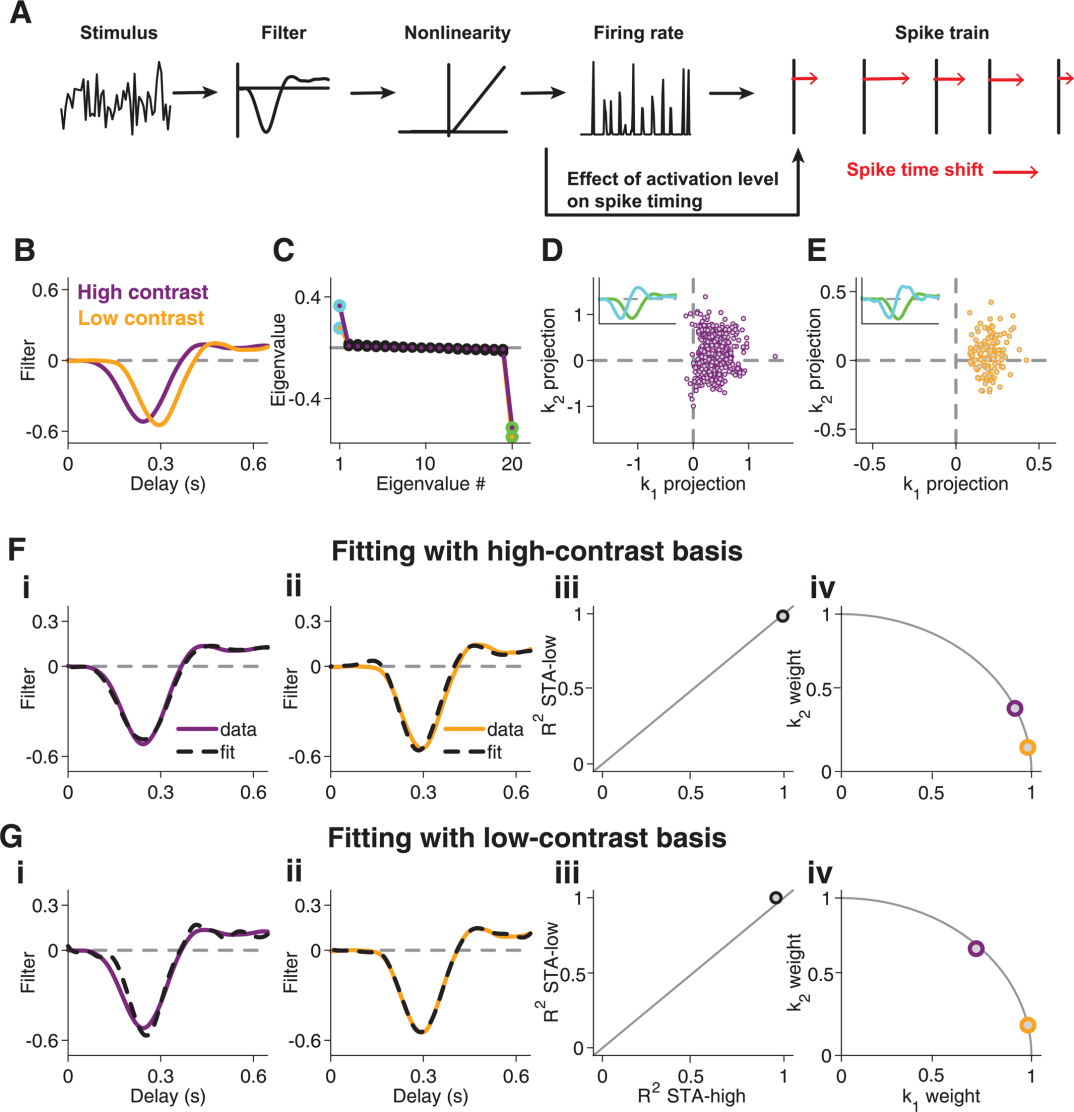
Contrast-dependent filter changes in a model with spike-timing dynamics. (**A**) Layout of the model. The stimulus feeds into a linear-nonlinear model, resulting in a firing rate, from which spikes are obtained by a Poisson process. Individual spikes are then shifted in time (as indicated by the red arrows), with the size of the shift depending on the activation level, which is given by the firing rate. (**B**) STAs obtained from model simulations for low and high contrast. (**C**) Eigenvalue spectrum obtained by STC analysis for high and low contrast, with the two most significant eigenvalues marked by green and light blue. (**D**) Scatter plot of spike-triggered stimuli for high-contrast stimulation of the model, projected onto *k*_1_ and *k*_2_. For clarity, only 0.2% of all analyzed spikes are shown in this plot. The inset shows the features *k*_1_ (green) and *k*_2_ (light blue), corresponding to the eigenvalues of the same color shown in (C). (**E**) Same as (D), but for low-contrast stimulation. (**F**) STA fits with features obtained from high-contrast STC analysis. Fit of high-contrast STA (**i**) and low-contrast STA (**ii**) as well as corresponding coefficients of determination (**iii**) and weights obtained for the two features (**iv**). (**G**) Same as (F), using the low-contrast-derived features.

The analysis of spike timing indicated that Type III cells display a particularly strong dependence of spike latency on activation level. To explore whether such spike timing dynamics could contribute to the contrast dependence of temporal filtering in these cells, we analyzed a simple model that included stimulus-dependent spike-time shifts, but no explicit contrast adaptation or negative feedback components. To do so, we generated spikes according to an LN model, followed by an inhomogeneous Poisson process (Fig 8A). Subsequently, we shifted the spike times, mimicking the spike-timing dependence observed in the data. Concretely, we used the output of the LN model as a measure of the activation level associated with each of the generated spikes. Depending on the activation level, each spike was then shifted to a later time, with smaller activation level corresponding to a larger shift (see Methods).

Analysis of spike trains obtained from this model revealed a slower STA under low contrast (Fig 8B) and a significant positive eigenvalue in the STC analysis (Fig 8C), with a continuous, elongated distribution of spike-eliciting stimuli in the space spanned by the identified features *k*_1_ and *k*_2_ (Fig 8D and 8E). All these characteristics are strikingly similar to the experimental data of Type III cells. Note that without the explicit spike-timing shifts, the applied LN model would only show a single negative significant eigenvalue. The positive eigenvalue of the model was thus indeed a product of the spike-time shift.

Finally, the STC-derived features *k*_1_ and *k*_2_ provided good fits of the model STAs (Fig 8F and 8G). In particular, the high-contrast STA was fitted well by the low-contrast basis (Fig 8Gi), which was a characteristic feature of TypeType III cells in the experimental data (cf. Fig 4D). This shows that the particularly strong spike-time shifts observed in Type III cells represent a viable model for explaining the specifics of temporal filtering and its contrast adaptation in these cells.

## Discussion

When visual contrast increases, responses of retinal ganglion cells become faster and more band-pass-like. This is typically described by contrast-induced changes in the shape of a temporal filter, which is traditionally measured as the spike-triggered average (STA) under temporal white-noise stimulation. Here, we explored alternatively whether contrast-induced changes in temporal filtering can be captured by a contrast-invariant set of filters, so that only the weights of the filters have to be adjusted when contrast changes.

The primary findings of this work are as follows: First, for most cells, a single basis of visual features indeed captured temporal filtering across contrast levels, and for the majority of these cells (here Type I cells), the multi-feature structure was found to be consistent with current models of contrast adaptation, based on activity-dependent gain control (Fig 5). Second, for certain On-Off-type cells (Type II cells), these adaptation dynamics were supplemented by separate adaptation in the On and Off pathway, with the latter showing stronger adaptation (Fig 6). Third, for a separate group of ganglion cells (Type III cells), the characteristics of the contrast dependence of temporal filtering were distinct from the other two groups and were not captured by common adaptation models. Instead, based on a separate analysis, we found that these cells have particularly strong stimulus-dependent latency shifts (Fig 7), which can explain the contrast-dependence of temporal filtering in these cells (Fig 8).

The systematic differences between different types of cells, revealed by the multi-filter analysis, shows that, despite the similarities of contrast-induced changes of the spike-triggered averages (Fig 1B), contrast adaptation is not an altogether homogeneous phenomenon across ganglion cells. Though it seems likely that all cells experience activity-dependent gain control and spike-timing dynamics to some degree, different phenomena appear to dominate in different cell types. While Type II cells seem to differ from Type I cells only in the addition of an independently adapting pathway, the strong dependence of spike timing on contrast and activation level set Type III cells apart from the other two types.

### Spike-triggered analysis

We based our analysis of the multi-filter structure of temporal filtering and its contrast dependence on spike-triggered covariance (STC) analysis, rather than on likelihood-based [50,51] or information-theoretic [22,34,52,53] alternatives because the STC presents a straightforward extension of the STA, which has commonly been used as a standard technique for analyzing contrast adaptation. In addition, STC analysis requires relatively few assumptions about the underlying stimulus-response relationship, whereas fitting the data with a more rigid modeling framework might have made it difficult to detect the particularly strong influence of spike-timing dynamics in a small subset of neurons.

Furthermore, we chose to perform the STC analysis separately for high and low contrast in order to test whether either contrast alone could deliver a subspace suited for generalization across contrast levels. The separate STC analyses turned out useful by revealing that Type III cells were specific in that the low-contrast basis fitted the high-contrast STA and that the number of relevant features did not increase with higher contrast. Using a simple white-noise stimulus and separately analyzing periods with different stimulus variance is a standard approach for assessing contrast adaptation [1–3,6,9]. An interesting future direction may be to generalize the contrast-induced changes to more complex stimuli with spatial structure [54] or with continuous variations of contrast over time. A low-dimensional description of the contrast-induced changes in temporal filtering as investigated in the present work may be a useful starting point for this endeavor.

A disadvantage of the STC approach is that there is no straightforward way of connecting the derived features to actual dynamics or mechanisms in the investigated system. In particular, the STC-derived features are constrained to be orthogonal to each other and bear significance primarily as a basis of the relevant stimulus subspace, which may alternatively be represented by any rotational transformation of this basis [15,34]. This is the reason why we did not compare the STC-derived features directly, but rather tested whether the STAs from both contrast conditions are contained in the same two-dimensional subspace. In principle, one might aim at identifying a more easily interpretable basis of the relevant subspace through subsequent analyses that incorporate appropriate prior assumptions, such as locality [55,56] or independence [57] of features. Yet, while locality seems an obvious assumption for spatial features, it is less clear which prior assumptions should guide the search for an appropriate feature basis of temporal filtering. Alternatively, one could apply a concrete model framework based on multiple filters and fit it to data via STC [38] or other methods [58,59]. Instead, we here chose to compare the STC analysis of the experimental data to the same analysis for different models to provide a qualitative comparison rather than aiming at improved interpretability of individual features.

### Potential mechanisms

A central mechanism of contrast adaptation in the retina is synaptic depression at bipolar cell terminals [5,9,12,13]. Indeed, our findings for Type I cells, which constituted the vast majority of cells in our recordings, were nicely reproduced by a kinetic model [37] that is well matched to the biophysics of vesicle depletion. In addition, spike generation contributes to contrast adaptation in salamander retinal ganglion cells through inactivation of sodium channels [10]. Such spike-dependent gain control is captured by the spike-feedback model.

Phenomenological models similar to the ones considered here have also been used to include stimulus-driven feedforward suppression [7,60–65]. Schwartz et al. [38], for example, showed for a sample ganglion cell that a model with suppressive stimulus features, derived via an STC analysis, can capture the cell’s contrast-induced changes in the STA. In the retina, a typical source of stimulus-driven suppression is feedforward inhibition. Yet, inhibitory mechanisms alone cannot account for contrast adaptation, as the adaptive effects have been shown to persist under pharmacological blockade of inhibition in the retina [9,54,66].

While we cannot exclude that alternative gain-control models might provide a good match for the results of Type II and Type III cells, specific observations led us to consider other dynamics for these cells. For Type II cells, the relevant ingredient was suggested by the clustered structure of the spike-triggered stimuli, indicating that convergence of On and Off pathway signals is relevant. Indeed, these cells typically showed responses to both increments and decrements of light intensity, yet this property was shared by a considerable fraction of cells from the other two types (Fig 2E). This is not surprising, as On-Off response characteristics by ganglion cells is a common feature of the salamander retina [23,26]. Type II cells are thus not an exclusive class of On-Off cells, but rather appear to correspond to cells with relatively pronounced On-type excitation under white-noise stimulation. In addition to the effects of On pathway signals, the Off pathway of Type II cells showed the contrast-adaptation characteristics of Type I cells. The need to separate spikes from the On and Off pathway for further analyzing Type II cells suggests that contrast adaptation occurs independently in each of these pathways. The simplest explanation is that a major component of contrast adaptation occurs before On and Off signals converge inside the ganglion cell, consistent with the implied role of synaptic depression between bipolar cells and ganglion cells.

Type III cells were distinct from the other two types in that they did not show emergence of additional features at higher contrast: the number of significant eigenvalues in the STC analysis did not differ between contrast levels, and the low-contrast STC analysis already provided features that captured temporal filtering even at high contrast. Instead, we found that these cells have a particularly strong dependence of spike timing on the activation level. Different mechanisms could contribute to the observed systematic latency shifts. For example, nonlinear gain control can effectively shift spikes forward in time for higher contrast by truncating the response at its tail end [67]. A similar scenario has been shown to occur in the spike-feedback model [36]: stronger activation leads to earlier threshold crossing, whereas later response parts are suppressed by the accumulation of negative feedback. Yet, this model alone produces a positive eigenvalue in the STC analysis only at fairly large levels of noise with spike jitter on the same time scale as the filter [20]. Another contribution could come from the temporal dynamics of spike generation itself. For example, models that describe spike generation through a saddle-node bifurcation are characterized by a strong dependence of spike delay on input current [68]. In biophysical terms, the weaker drive of a smaller stimulus allows slow inactivation processes, such as potassium currents or sodium-channel inactivation, to keep up with the cell’s depolarization and thereby create arbitrary long delays for sufficiently weak activation.

### Cell-type classification

The most basic classification of ganglion cells into subtypes distinguishes between On, Off, and On-Off cells, depending on the cells’ responses to steps in light intensity [69]. This scheme still represents the most fundamental cell-type classification also in the salamander retina [26], but alternative classification schemes have been suggested based on the analysis of the shapes of STAs [27,28,70] as well as based on the characteristics of multiple STC-derived features [20]. Yet, a clear segmentation of salamander retinal ganglion cells into distinct cell types or even a reliable estimate of the number of cell types is still lacking. In particular, tiling of receptive fields, which represents a hallmark of an identified cell type in the retina, has been observed in only few individual cases [28,71].

We here therefore followed a pragmatic course by classifying the analyzed ganglion cells into three broad classes based on the cells’ STC eigenvalue spectra, whose different structures are directly relevant for choosing the appropriate basis of the STA fits in this work. Clearly, some or all of these classes should be expected to contain multiple actual types of ganglion cells. The three classes also showed other differences than their STC spectra, though none of these clearly separated the groups. Type II cells had, on average, the strongest On-type responses under full-field steps in light intensity, and Type III cells tended to have slower filters than cells from the other two groups.

Fairhall et al. [20] previously investigated salamander retinal ganglion cells with STC analysis and described five cells types, though stating that this did not represent a rigorous clustering, but rather a suitable representation of a continuum of response types. Still, similarities between the five response types described by Fairhall et al. and our three types suggest a rough match: The filter-and-fire and complex filter-and-fire cells of Fairhall et al., which both had no positive eigenvalue and only differed in the number of negative eigenvalues, would thus correspond to our Type I cells. Furthermore, their bimodal cells showed two clusters of spike-eliciting stimuli with correspondence to On and Off stimuli and thus match our Type II cells. Finally, the Hodgkin-Huxley-like cells and the ring cells then correspond to our Type III cells, with a positive eigenvalue and a continuous distribution of spike-eliciting stimuli. Interestingly, Fairhall et al. observed particularly large spike-time jitter for their ring cells [20], matching another property of our Type III cells.

Recently, different types of short-term plasticity had been reported in the salamander retina, with some cells showing sensitization, described as elevated sensitivity for several seconds after strong stimulation [71,72]. Here, we did not distinguish between sensitizing and classically adapting cells because our analysis focused on temporal filtering during the steady state of a given contrast level, rather than on transient sensitivity changes. It also seems that the comparatively long periods between contrast switches and the smaller difference between the contrast levels in our study might trigger sensitization less effectively.

### Application to other systems

Similar analyses of how adaptation affects the multi-filter structure of sensory neurons have previously been performed in the somatosensory [73,74] and the auditory system [75]. Neurons in the somatosensory barrel cortex were found to be selective to multiple features under white-noise whisker stimulation. Changes in stimulus variance were then shown to lead to a gain change for each feature, but the features themselves remained invariant, and the gain change was approximately equal for all features [73]. Changes in the correlation structure of multi-whisker stimulation, on the other hand, altered the relative contributions of the features [74]. Likewise, for auditory neurons in songbirds, changes in variance of acoustic stimuli left the relevant features invariant, but changed the relative importance of the features [75], similar to our observations in the retina. Changing the mean stimulus intensity, on the other hand, fundamentally altered the features of the auditory neurons [75], indicating different functional roles of adaptation to mean and variance in this system.

Together with the present work, these studies indicate that investigating adaptation phenomena from the perspective of multiple parallel filters can be a fruitful endeavor. The occurrence of multiple relevant stimulus features appears to be a ubiquitous finding, including, besides the retina, the downstream visual system [39,76–82], as well as other sensory systems, such as auditory [75,83,84], somatosensory [73,74,85], olfactory [86,87], and mechanosensory [88]. Furthermore, adaptive changes in sensory processing are found throughout different sensory systems. In particular, adaptation to stimulus variance in different auditory systems [75,89–93] and in the somatosensory system [73,85,94] as well as adaptation to luminance [95,96] and to other stimulus correlations [97,98] in the early visual system bear striking similarity to retinal contrast adaptation. Thus, the question whether adaptation alters the different stimulus features themselves or merely their relative contributions to signal processing pertains to a wide range of systems.

## Methods

### Ethics statement

All experimental procedures were performed in accordance with national and institutional guidelines and approved by the institutional animal care committee of the University Medical Center Göttingen (protocol number T11/35).

### Electrophysiology and visual stimulation

Retinas were obtained from dark-adapted adult axolotl salamanders (Ambystoma mexicanum; pigmented wild type) of either sex. After enucleation of the eyes, retinas were isolated from the eyecup and cut in half. One retina half was placed ganglion-cell-side-down on a planar multielectrode array (Multichannel Systems, 252 channels, 10-μm electrode diameter, 60-μm spacing) for extracellular recording, while the other retina pieces were stored in oxygenated Ringer’s solution (110 mM NaCl, 2.5 mM KCl, 1.6 mM MgCl2, 1.0 mM CaCl2, 22 mM NaHCO3, 10 mM D-glucose, equilibrated with 95% O2 and 5% CO2) for later recording. The preparation was performed with infrared illumination under a microscope equipped with night-vision goggles. During recordings, retinas were continuously perfused with the Ringer’s solution at room temperature (20°C-22°C). The measured voltage signals were amplified, band-pass filtered between 300 Hz and 5 kHz, and digitized at a sampling frequency of 10 kHz. Potential spikes were detected by threshold crossing. Separation from noise and sorting into units representing individual ganglion cells was achieved by custom-made software, based on a Gaussian mixture model and an expectation-maximization algorithm [99]. Only well-sorted units with a clear refractory period were used for further analysis.

Data are available from the Dryad Digital Repository (doi: 10.5061/dryad.7r7n7).

To visually stimulate the retina, the screen of a gamma-corrected miniature OLED monitor (eMagin, OLED-XL series, 800x600 pixels) was focused through a telecentric lens onto the photoreceptor layer. The projection of the screen covered the recorded piece of retina. The size of individual image pixels on the retina was 7.5 μm x 7.5 μm. The stimulus screen was updated with a frame rate of 60 Hz and controlled through custom-made software, based on Visual C++ and OpenGL.

Visual stimuli for the contrast-adaptation analysis were composed of a spatially uniform flicker of light intensity around a mean intensity of *M* = 39.5 mW/m² in the photopic range. New intensity values were drawn randomly from a Gaussian distribution with standard deviation *σ* at a rate of 30 Hz. The contrast level *C = σ/M* alternated between 100-sec episodes of low contrast (*C* = 12%) and 20-sec episodes of high contrast (*C* = 32%). For each experiment, we typically recorded a total of 120–240 such episode pairs of high and low contrast.

To measure responses to steps in light intensity, we used 5–10 min of alternating 2-sec periods of bright and dark illumination at 100% contrast around the same mean illumination as for the flicker stimulation. To calculate average firing rates, spikes were counted over the duration of each illumination level, excluding the first 100 ms after each switch in illumination.

### Spike-triggered analysis

For each recorded ganglion cell and both contrast conditions, the spike-triggered average (STA) was computed [31,100] and compared to the relevant features obtained from an eigenvalue analysis of the spike-triggered covariance (STC) matrix [15–19]. STC analysis provides a straightforward extension of the STA, which has been commonly used to characterize contrast adaptation. Furthermore, this analysis allowed us to extract multiple relevant stimulus features for assessing temporal filtering without having to assume a particular model of the origins of these features or their interactions.

For both spike-triggered analyses, the stimulus sequence *s*(*t*) was defined as the sequence of contrast values, corresponding to the deviations from mean light intensity, normalized to unit standard deviation, and sampled at the stimulus update rate of 30 Hz. Spike trains were binned at the same sample rate. We used spikes from the entire span of each contrast episode (100 sec for low contrast, 20 sec for high contrast). This decision is justified by the fact that the investigated changes in temporal filtering are known to occur immediately after a switch in contrast [1], whereas sensitivity of the ganglion cells changes more slowly, as reflected by changes in the average firing rate over several seconds after a contrast switch. In order to verify that these non-stationary effects of sensitivity do not affect our results, we also performed the entire analysis with only the second half of each contrast episode, where sensitivity is nearly stationary. This added noise to the estimated filters because of the reduced data but otherwise had no effect on the results (S1 Fig).

To calculate the STA and STC, we collected for each spike time *t*_*n*_ the stimulus sequence *s*_*n*_(*τ*) = *s*(*t*_*n*_ − *τ*) that preceded the spike, where the lag *τ* covers 20 time bins. The STA is then obtained as the average over all spikes, $\bar{s}(\tau )={\langle s_{n}(\tau )\rangle }_{n}$. In practice, if there is more than one spike in a time bin, the corresponding stimulus sequence preceding that bin is multiplied by the number of spikes in the bin. STC analysis is based on the covariance matrix *C* of spike-triggered stimulus sequences, $C_{\tau_{1},\tau_{2}}={\langle \left[s_{n}(\tau_{1})-\bar{s}(\tau_{1})][s_{n}(\tau_{2})-\bar{s}(\tau_{2})\right]\rangle }_{n}$. We subtracted the prior covariance matrix of all stimulus sequences, *C*_*prior*_, which here is just the identity matrix because of the white-noise statistics of the stimulus. From the resulting matrix Δ*C* = *C* − *C*_*prior*_, relevant features are found by diagonalizing Δ*C* to obtain its eigenvalues and corresponding eigenvectors. In essence, this analysis is similar to a principal component analysis of the set of spike-triggered stimulus sequences, except for the subtraction of the prior covariance matrix, which shifts the baseline of eigenvalues to zero and allows for the occurrence of negative eigenvalues. Significant deviations of eigenvalues from the baseline indicate that the corresponding eigenvectors represent relevant visual features. To compare shapes of the obtained temporal filters from STA and STC analysis, all filters were normalized so that the sum of squared filter components equaled unity. For plotting filters, we upsampled all filters from the original 20 sample points to 96 sample points by cubic spline interpolation.

To assess eigenvalue significance, we applied a spike-shuffling technique [15,17] by randomly time-shifting individual spikes within their corresponding stimulus episode of either high or low contrast and performing the STC analysis with the shifted spike train to get an eigenvalue spectrum that is determined by sampling noise. After repeating the time-shift analysis 1,000 times, we estimated the 95% confidence interval for the eigenvalue spectrum and compared it to the true eigenvalue spectrum. If the maximal or minimal eigenvalue lay outside the confidence interval, it was regarded as significant. This analysis was then repeated in a nested fashion [15] by projecting out the corresponding significant eigenvector from all stimulus sequences and repeating the analysis in the reduced stimulus space to test for further significant eigenvalues. This procedure was iterated until both the maximal and minimal eigenvalue of the remaining spectrum lay inside the confidence interval.

We computed nonlinearities corresponding to individual filters by a histogram method [31]. To do so, stimuli were first convolved with the respective temporal filters to obtain a generator signal. This generator signal was then binned into 40 bins in a way so that each bin contained the same number of data points. We displayed the nonlinearity by plotting the average generator signal against the average spike rate for each bin.

### Information encoded by filters

We evaluated the relevance of different features by computing the information transmitted by a feature *k* about individual spikes [101] according to *I*_feature_(*k*) = ∫ *ds P*(*s* | spike)log_2_(*P*(*s* | spike) / *P*(*s*)), where *s* represents the projections of the stimulus sequences onto the feature *k*, *P*(*s*) is the probability distribution of the prior stimulus ensemble along the direction *k*, and *P*(*s* | spike) is the probability distribution of spike-triggered stimuli along this direction. The integral was evaluated by discretizing the projected stimulus values *s* with a bin size equal to 0.1 of the stimulus standard deviation. We also computed the information captured by two features *k*_1_ and *k*_2_ with the same formula by replacing the distributions of *s* with the two-dimensional distributions over the two corresponding stimulus projections *s*_1_ and *s*_2_ [101].

For a subset of the recorded cells, we also measured the total information transmitted by single spikes [101]. To do so, we used a stimulus where the same white-noise sequence of 100 sec at low contrast and 20 sec at high contrast was repeated for 50 trials or more to obtain the time-varying firing rate *r*(*t*). We then computed the total single-spike information as $I_{\text{one spike}}=\frac{1}{\bar{r}T}\int_{0}^{T}\;dt\;r(t)\;{\text{log}}_{2}\frac{r(t)}{\bar{r}}$ where $\bar{r}$ is the mean firing rate and *T* is the stimulus duration. The integral was evaluated by discretizing time according to the stimulus update rate.

All information values were corrected for bias that arises from finite sampling by analyzing subsections of the data (80–100%) and using linear extrapolation to estimate the information value at infinite sample size [101,102].

The primary use of the information-theoretic analysis in this work was to order the features obtained from the STC analysis according to their information content and to provide an absolute measure of how relevant the features were. In particular, we used this analysis to classify the ganglion cells. We calculated the information for each eigenvector *v*_*i*_ of the STC analysis, with *v*_*i*_ denoting the eigenvector of the *i*-th largest eigenvalue (*i* = 1,…, 20). To reduce the effect of noisy cells on our population analysis, we discarded cells that had no eigenvector with an information rate of at least 0.8 bits/spike or for which the two most informative eigenvectors did not lie at the ends of the eigenvalue spectrum, for example, if the most informative feature was *v*_20_, but the second-most one was neither *v*_19_ nor *v*_1_. We thereby discarded 98 out of 443 recorded cells. For further analysis, we selected those two eigenvectors with maximal information transmission as the visual features *k*_1_ and *k*_2_. These then always corresponded to a set of two of the features *v*_1_, *v*_19_, and *v*_20_, with *v*_*i*_ denoting the eigenvector of the *i*-th largest eigenvalue (*i* = 1,…, 20). To distinguish between cell types, we thus focused our analysis on how the cells were distributed in the space spanned by information values corresponding to *v*_1_, *v*_19_, and *v*_20_, as discussed in the Results section.

### Fitting STAs with multiple features

To fit an STA by visual features derived from the STC analysis, we subdivided the data for each contrast condition into a training set (80% of the contrast episodes, randomly chosen) to obtain the STC-derived features and a test set (the remaining 20% of the episodes) to obtain the STA. The STA $\bar{s}(\tau )$ was then fitted by a linear combination of the two features *k*_1_ and *k*_2_: $\hat{s}(\tau )=\alpha_{1}\cdot k_{1}(\tau )+\alpha_{2}\cdot k_{2}(\tau )$. The weights *α*_1_ and *α*_2_ were optimized according to a least-squares criterion. The goodness of fit was quantified by the coefficient of determination *R* ^2^, based on the STA $\bar{s}(\tau )$ and its fit $\hat{s}(\tau )$: $R^{2}=1-\frac{\sum_{\tau }{\left(\bar{s}(\tau )-\hat{s}(\tau )\right)}^{2}}{\sum_{\tau }{\left(\bar{s}(\tau )-\langle \bar{s}(\tau )\rangle \right)}^{2}}$, where 〈⋅〉 denotes the average over time.

For Type II cells, we also obtained an alternative basis for fitting the STAs by identifying filters *k*_*ON*_ and *k*_*OFF*_, which represent signal processing by the On and Off pathway of the retina, respectively. These filters were computed by subdividing the spike-triggered stimuli according to whether they corresponded to the On or Off pathway. Specifically, all spike-triggered stimuli were projected onto *k*_1_ and *k*_2_ and separated in this two-dimensional space into two clusters by k-means clustering. The average spike-triggered stimulus segment of each cluster then yielded the On and Off filters *k*_*ON*_ and *k*_*OFF*_, respectively [20,23,24,48].

To assess how much of the contrast-induced variations of the STAs were captured by each feature basis, we quantified how well the difference between the high-contrast and the low-contrast STA was captured by the differences of their fits. To do so, we computed the coefficient of determination for the STA difference. Concretely, based on the STAs ${\bar{s}}_{\text{high}}(\tau )$ and ${\bar{s}}_{\text{low}}(\tau )$ obtained under high and low contrast and of the corresponding fits ${\hat{s}}_{\text{high}}(\tau )$ and ${\hat{s}}_{\text{low}}(\tau )$ obtained with a single basis, we computed the coefficient of determination as $R^{2}=1-\frac{\sum_{\tau }{\left[({\bar{s}}_{\text{high}}(\tau )-{\bar{s}}_{\text{low}}(\tau ))-({\hat{s}}_{\text{high}}(\tau )-{\hat{s}}_{\text{low}}(\tau ))\right]}^{2}}{\sum_{\tau }{\left[({\bar{s}}_{\text{high}}(\tau )-{\bar{s}}_{\text{low}}(\tau ))-\langle {\bar{s}}_{\text{high}}(\tau )-{\bar{s}}_{\text{low}}(\tau )\rangle \right]}^{2}}.$

### Estimating spike-timing dynamics

The delay of a spike after the spike-eliciting stimulus (i.e., the spike latency) as well as its timing variability (spike jitter) can depend on the level of activation of a cell and thereby affect temporal filtering as assessed by spike-triggered analyses [41–43]. In order to compare such spike-timing dynamics for different ganglion cell types under white-noise stimulation, we estimated the dependence of spike timing on the activation level in the following way: for each cell and each contrast level, we filtered the white-noise stimulus with the corresponding STA and used the resulting filter output as an estimate of the activation level. We then identified all local maxima of this filter output and binned them according to their peak value into 40 bins so that each bin contained the same number of local maxima. For each bin, we collected the spike trains in a window of 367 ms around the local maxima. Averaging these spike trains provided us with a histogram of spike probabilities at different times relative to the local maximum of the filter output. Histograms for small peak values of the filter output suffered from noise, owing to the paucity of contributing spikes. We therefore restricted further analysis to the histograms corresponding to the highest activation levels, using ten and four histograms for the high-contrast and low-contrast stimulus, respectively. We then normalized the histograms to their peak values in order to analyze their shapes independently of the number of contributing spikes.

For each spike-probability histogram, the position of its peak was obtained from fitting a 2nd-order polynomial through its largest data point and the two neighboring data points. The peak position yielded a characteristic spike-time shift for the corresponding activation level. We found that the relation between activation level and characteristic spike-time shift over the analyzed range of activation levels could be well fitted by a straight line. We therefore used the slope of this line, ∆Spike-time shift/∆Activation level (Fig 7D), as a measure of the sensitivity of spike-timing on activation level.

### Modeling

We explored two computational models of ganglion cell activity that had previously been shown to capture essential aspects of contrast adaptation in these cells, in particular with respect to the temporal filtering characteristics of the cells. As stimuli for both models, we used Gaussian white-noise sequences, similar to the experiments, sampled in discrete time steps of ∆t = 33.3 ms, with zero mean and standard deviation 0.32 for high contrast and 0.12 for low contrast, respectively.

The first model [35,36] is based on a temporal filter of the stimulus, followed by a threshold operation to determine spikes. In addition, a feedback signal is subtracted from the feedforward filter signal before application of the threshold. Each spike increments the feedback signal by a fixed amount. In between spikes, the feedback decays exponentially with a given time constant. For the temporal stimulus filter, we used the experimentally measured STA, normalized to unit power, of a sample ganglion cell at low contrast. (Note, though, that the model did not aim at reproducing the particular responses of the sample cell. Rather, we aimed at analyzing the generic behavior of the model, based on parameters that matched the time scales of our data.) The choice of the other model parameter values was guided by the values used in [36], but using slower feedback decay to account for the relatively slow filter time scales of the salamander retina. Specifically, we used a threshold of 0.2, a spike-triggered increase of the feedback signal by 3.0, and a time constant for the decay of the feedback signal of 250 ms.

The second model [37], called linear-nonlinear-kinetic (LNK) model, uses a sequence of linear stimulus filtering, nonlinear transformation, and a biophysically plausible first-order kinetic process to transform the stimulus into the membrane potential of a ganglion cell. Concretely, for the first two stages of the model, we used the same temporal filter as for the spike-feedback model, combined with the sigmoidal nonlinearity of the form 1 / (1 + exp(−*a*(*x* − *θ*))) with *a* = 2.5, *θ* = 2. The kinetic stage consisted of a resting state, an active state, and two inactive states, with transition rates between them. The value of the active state corresponds to the model’s output. As an input to the kinetic stage, the signal after the nonlinearity, termed *u*(*t*), modulates both the transition from the resting state to the active state as well as the transition from the first to the second inactive state. The transition rates between the states were slightly modified as compared to [37] to make recovery of adaptation slower because of the comparatively slower filters in our data. The applied values are indicated in the corresponding figure in the Results section. For both models, we also tested variations of the parameter values over some range and found that the obtained results and conclusions are robust to these variations.

To selectively explore the effect of spike-timing dynamics on STC analysis and contrast-induced changes in temporal filtering, we also investigated a simple extension of an LN model, with added systematic spike-timing shifts, but no activity-dependent gain control. First, we implemented an LN model by again using the same stimulus structure and temporal filter as in the other two models. We then applied a threshold-linear transformation *a*[*x* − *θ*]_+_ with *a* = 20 Hz and *θ* = 0.08, and determined spikes from the resulting firing rate according to an inhomogeneous Poisson process. Finally, each spike was delayed by an amount that depended on the value of the firing rate when the spike had been triggered. Specifically, we linearly divided the range of firing rates from zero to 15 Hz into four levels. Firing rates larger than 15 Hz were treated the same as those in the largest of these four levels. For each level *L* (*L* = 1,…, 4, with larger *L* corresponding to larger firing rates), we shifted the corresponding spikes by 4 − *L* units of the time step ∆t. Thus, spikes that had been generated (by chance) when the firing rate was actually small experienced a larger delay than spikes that had been generated by a higher firing rate.

## Supporting Information

S1 Fig Control analysis, using only the second half of each contrast episode. Here, the spike-triggered analyses were performed by ignoring all spikes in the first half of each contrast episode in order to control for the effect of the early transient changes in firing rate. Results are shown for three sample cells (A-F, same cells as shown in Fig 1 of the main text) as well as for the STA fits of all recorded cells (G, H). Panels A-E should be compared with Fig 1 from the main text, panel F with the first column of Fig 3, and panels G,H with Fig 4A and 4D. As expected, extracted filters and fits show a higher level of noise as compared to including spikes from the entire duration of the contrast episodes, but otherwise, results and conclusions are unaffected.
(EPS)
